# Review of Microbottle Resonators for Sensing Applications

**DOI:** 10.3390/mi14040734

**Published:** 2023-03-26

**Authors:** Huda Adnan Zain, Malathy Batumalay, Hazlihan Haris, Ismail Saad, Ahmad Razif Muhammad, Siti Nasuha Mustaffa, Arni Munira Markom, Hazli Rafis Abdul Rahim, Sin Jin Tan, Sulaiman Wadi Harun

**Affiliations:** 1 Department of Electrical Engineering, Faculty of Engineering, University of Malaya, Kuala Lumpur 50603, Malaysia; 2 Faculty of Data Science and Information Technology, INTI International University, Nilai 71800, Negeri Sembilan, Malaysia; 3 Faculty of Engineering, Universiti Malaysia Sabah (UMS), Kota Kinabalu 88400, Sabah, Malaysia; 4 Institute of Microengineering and Nanoelectronics (IMEN), Universiti Kebangsaan Malaysia (UKM), Bangi 43600, Selangor, Malaysia; a.razif@ukm.edu.my (A.R.M.); p128826@siswa.ukm.edu.my (S.N.M.); 5 School of Electrical Engineering, College of Engineering, Universiti Teknologi MARA, Shah Alam 40450, Selangor, Malaysia; 6 Faculty of Electronic and Computer Engineering, Universiti Teknikal Malaysia Melaka, Melaka 76100, Melaka, Malaysia; 7 School of Engineering, UOW Malaysia KDU University College, Shah Alam 40150, Selangor, Malaysia

**Keywords:** microbottle resonators, sensing, optical resonators, whispering-gallery modes

## Abstract

Microbottle resonators (MBR) are bottle-like structures fabricated by varying the radius of an optical fiber. MBRs can support whispering gallery modes (WGM) by the total internal reflection of the light coupled into the MBRs. MBRs have a significant advantage in sensing and other advanced optical applications due to their light confinement abilities in a relatively small mode volume and having high Q factors. This review starts with an introduction to MBRs’ optical properties, coupling methods, and sensing mechanisms. The sensing principle and sensing parameters of MBRs are discussed here as well. Then, practical MBRs fabrication methods and sensing applications are presented.

## 1. Introduction

**Figure 1:**
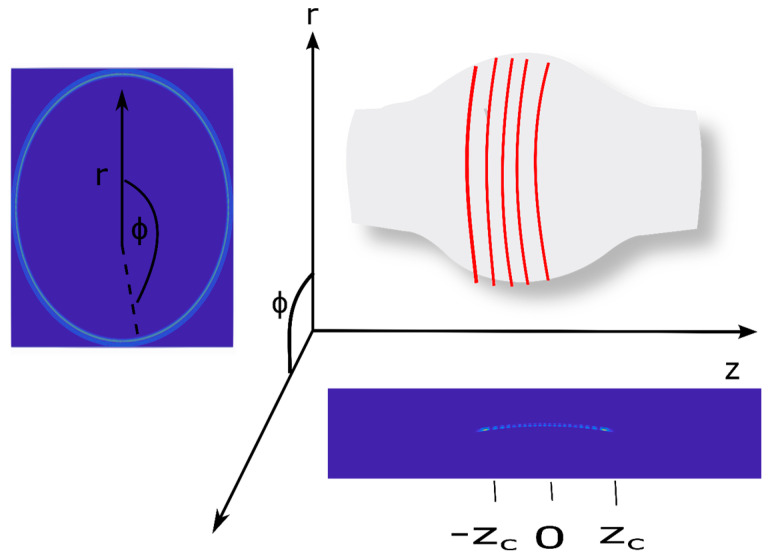
Microbottle resonators’ light confinement.

Microbottle resonators (MBR) have an azimuthally symmetric and axially extended shape. MBRs confine light through the whispering gallery modes and the bouncing-ball confinement effects [1] as shown in Figure 1. MBRs’ optical states are localized inside the resonator by total internal reflection and have whispering gallery mode structures such as the acoustic modes reported by Lord Rayleigh.

The unique properties of MBRs are their bottle-like shape and dimensions and their 3D light confinement. The shapes of microspheres and microbottles differ due to the ratio between the azi-muthal and axial radii. While the shapes of microspheres are comparable to those of other whispering gallery mode (WGM) resonators, the axial radius of MBRs is much greater than their azimuthal radius, making it easier to access the WGM from the sur-rounding medium of the MBRs. Thus, MBRs are ideal for many advanced applications, such as lasing [2], add-drop filters [3], and sensors [4].

MBRs are resonators shaped like a bottle or a spheroid after elongation. MBRs are formed by introducing radius variations to fiber. In 2004, the concept of confining light by thickening a cylinder’s radius was introduced [5]. The structure was called a microbottle to emphasize its geometric similarities to magnetic bottles confining hot plasmas.

MBRs were used as add-drop filters due to their tunable characteristics. It was shown that the position of the excitation and probe fibers affects the resonators’ free spectral [3]. In 2018, cascaded MBRs consisting of partially overlapping bottle shapes were used as add-drop filters and showed stable responses with quality factors that can reach 10^6^ [3].

A tunable micro-laser had an erbium-doped hybrid microbottle cavity coated with iron oxide nanoparticles. The structure showed a Q factor of about 5.2 × 10^7^ in the 1550 nm band. A lasing threshold of 1.65 mW was achieved with a non-resonant pump of 980 nm [6]. A hybrid MBR with iron nanoparticle coatings showed Q factors over 108. The MBR reached the Brillouin and Raman lasers with thresholds of 0.17 mW and 0.25 mW, respectively, and wavelength tuning of the Brillouin laser with a range of 2.68 nm and the Raman laser with a range of 2.32 nm [7]. MBRs are versatile tools for many applications. In the next section, we discuss the optical properties of optical resonators. These properties help describe the ability of MBRs to confine light.

## 2. MBRs Optical Properties

A WGM resonator, such as an MBR, can be described by optical properties such as resonance wavelength, quality factor, mode volume, FSR, and finesse.

### 2.1. Resonant Wavelength

The light circulating inside the resonator can experience constructive interference at specific resonance wavelengths, resulting in resonance at certain wavelengths. This constructive interference only happens to the circulating light waves when the total propagation distance is an integer multiple of the resonance wavelength. These resonance wavelengths can be determined using

(1) $$m\lambda =2\pi Rn_{eff}.$$

Given that m is an integer azimuthal mode number, R is the radius of the WGM resonator, λ  is the resonant wavelength, and $n_{\mathrm{eff}}\;$is the effective refractive index of the WGM resonator [8]. Tracking shifts in resonance wavelengths is a common sensing principle of MBR sensors.

### 2.2. Free Spectral Range (FSR)

The distance between two consecutive WGM resonances is the free spectral range of the resonator. Alternatively, a resonator’s free spectral range is the distance between adjacent resonant angular mode numbers (m, m − 1). For the fundamental modes of a WGM with the radial mode number r = 1:

(2) $$2n\pi R=m\lambda_{m}=\left(m-1\right)\lambda_{m-1}=\left(m-1\right)\left(\lambda_{m}+\;\Delta \lambda \right),$$

with R as the WGM cavities’ radius, *n* as the refractive index of the resonator, and $\lambda_{m}$/$\lambda_{m-1}$ as the two adjacent modes’ wavelengths. For large modes (large m), the free spectral range can be assumed to be constant. The formula simplifies to

(3) $$\Delta \lambda =\frac{\lambda_{m}{}^{2}}{2n\pi R}.$$

TFSR depends on the size of the WGM resonator. Larger WGM resonators have denser transmission spectra and smaller FSR. Smaller WGM resonators have a larger FSR and wide spaces between resonances. A larger FSR can increase the resonators’ dynamic detection range and help obtain single-mode laser output output [9,10,11].

### 2.3. Mode Volume

Smaller mode volume (V) is an advantage of using MBR. The mode volume is the localization of the electromagnetic field in a WGM resonator. Mode volume is the ratio between the full space integral for the energy density of the mode field and the maximum energy density.

(4) $$V=\frac{\int n^{2}\left(r\right){\left|\vec{E}\left(r\right)\right|}^{2}d^{3}r\;}{\max\left(n^{2}\left(r\right){\left|\vec{E}\left(r\right)\right|}^{2}\right)}.$$

Given that the point energy density is $n^{2}\left(r\right){\left|\vec{E}\left(r\right)\right|}^{2}$ and the electric field vector is $\vec{E}\left(r\right)$. The energy density of the WGM inside the resonator and the mode volume are inversely proportional. Larger energy density means smaller mode volume and stronger light and matter interaction. Thus, smaller mode volumes can result in higher sensitivity of MBR to the surrounding medium. The mode volume is an important parameter in nonlinear applications [11,12,13]. 

### 2.4. Quality Factor

The quality factor shows the WGM resonators’ ability to store energy at specific frequencies. The quality factor is used to analyze the ability of an MBR or WGM resonator to trap light energy inside it for a given duration. Larger quality factors mean longer lifetimes. Quality factors can be analyzed in the time or frequency domain [14].

(5) $$Q_{{}^{\circ}}=2\pi \frac{E_{stored}}{P_{diss}}=\omega \tau =\frac{\omega }{\Delta \omega }=\frac{\lambda }{FWHM},$$

with ω as the resonance frequency,${\;E}_{\mathrm{stored}}\;$as the stored energy in the resonator, $P_{\mathrm{diss}}$ as the dissipated power, and the lifetime of the photons as τ. The resonance linewidth and FWHM can be obtained from the frequency spectrum measurements. The quality factor is defined as the ratio between the resonance wavelength and the FWHM. Different materials have different Q factors [14].

The total quality factor of coupled resonators is the sum of the intrinsic quality factor and the additional Q factor due to coupling and external mode losses.

(6) $$Q_{total}^{-1}==Q_{{}^{\circ}}^{-1}+Q_{external}^{-1}.$$

The intrinsic factor ($Q_{{}^{\circ}}$) for an uncoupled WGM resonator is

(7) $$Q_{{}^{\circ}}^{-1}=\sum Q_{i}{}^{-1}=Q_{radiation}^{-1}+Q_{material}^{-1}+Q_{contsamination}^{-1}+Q_{scattering}^{-1}.\;$$

$Q_{radiation}$ is the radiative loss caused by energy escaping the WGM surface as the evanescent field. It is an intrinsic parameter that is controlled by the curvature of the WGM resonator. Smaller cavities with larger curvatures can cause the resonance wavelength to undergo less total internal reflection, resulting in a steeper incidence angle and reducing the light confinement ability of the WGM resonator. Radiative losses are the ratio of the real and imaginary wave numbers [8,14,15].

$Q_{material}$ is related to the Rayleigh scattering and the WGM material absorption. $Q_{material}$ can be approximated as

(8) $$Q_{material}=\frac{\;2\pi n}{\alpha \lambda }\;,$$

with *n* as the refractive index of the WGM resonator material and α as the linear absorption coefficient.

$Q_{scattering}$ shows the scattering losses caused by the residual surface inhomogeneities/roughness.

(9) $$Q_{scattering}=\frac{\lambda^{2}R}{\pi^{2}{B\sigma }_{\mathrm{rms}}^{2}}.$$

The root-mean-squared size is $\sigma_{rms}$, and the roughness correlation length is B. Thus, the scattering quality factor is directly proportional to the resonators’ radius underneath the irregularities on the resonator’s surface. Bigger resonators are better at storing light. Minimizing the size of irregularities can increase the Q factor. Polishing and drying in the post-processing stage can lower scattering losses. Selecting optimal materials that are amorphous, homogenous, and isotropic can also minimize the losses [9].

${\;Q}_{\mathrm{contamination}}^{-1\;}$ is the loss caused by contamination on the surface of the WGM resonator, such as water molecules or oxidized coating [9]. Q factors in the 10^3^–10^6^ range are considered high. Q factors larger than 10^6^ are ultra-high. Higher Q factors mean greater energy densities and longer lifetimes for light–matter interactions [9].

### 2.5. Finess

Finesse estimates the bounces the light takes inside a WGM resonator during its lifetime before light’s absorption or leakage to the medium outside the resonator. Finesse is the ratio of the free spectral range to the resonance linewidth (FWHM), and it is the distance between consecutive resonances measured in linewidth terms. Finesse can be calculated by multiplying the quality factor with the ratio of the free space spectral range to the spectrum [9].

(10) $$F=\;\frac{\Delta \omega_{FSR}}{\Delta \omega_{FSRM}}=Q\;\frac{\Delta \omega_{FSR}}{\omega_{{}^{\circ}}}=Q\frac{\lambda }{2\pi nR}.$$

## 3. MBR Light Coupling

**Figure 2:**
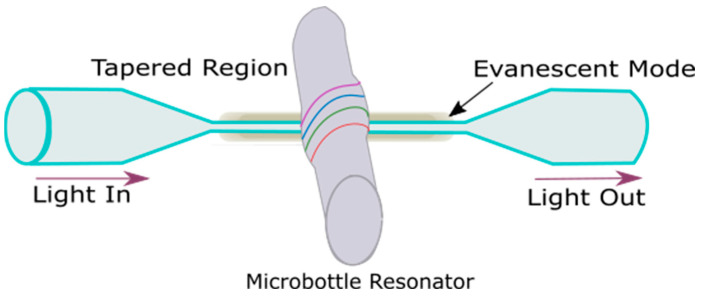
MBR coupling method; tapered fiber coupling.

Light needs to be coupled into an MBR cavity. Light can be coupled using evanescent waves or launched directly into the MBR element, with the resulting scattered light then collected using optical fiber or lenses. Coupling efficiency varies depending on the coupling method used. The coupling efficiency is the ratio of the MBR coupled power to the input power. The coupling principle of MBRs is similar to that of other WGM resonators. Figure 2 shows some coupling methods for MBR.

### 3.1. Phase Matching

Phase matching is needed to obtain the highest possible efficiency from the coupling. For a resonator, phase matching is a state when the propagation constant of the WGM cavity is the same as the propagation constant of the light mode [12,15,16]. The distance between the resonator and the light coupler should be ideal for optimal coupling. This distance should be optimized to compensate for the resonator losses caused by the power coupling. Critical coupling is achieved when the coupler output reaches zero at the resonant wavelength [8].

### 3.2. Free-Space Coupling

The first developed method for WGM light coupling was free-space coupling. It directly couples light with the WGM element. When light is directed at the WGM element and the frequencies of the incident light and the WGM element’s resonant frequency match, a part of the incident light couples into the WGM element, and the other part of the incident light is scattered. If a photodetector collects the scattered light, it can be analysed to study its spectrum and intensity. Usually, a spatial optical path is needed. A focusing lens directs light towards the edge of the WGM element. A collimated mirror sends the scattered light to the photodetector [17,18].

This method has a low efficiency of 20–30%. This low efficiency is due to the relation between the WGM cavity size and radiation intensity [18]. Large WGM elements have dimensions larger than the incident light, so radiation weakens. This method does not need high-quality WGM elements or waveguides for the light. It also works well with visible light. However, the low efficiency and the need for small-sized WGM elements limit the use of this method [17].

### 3.3. Evanescent Waves Coupling

Evanescent waves can be coupled into a WGM element in multiple ways, including prism coupling, fiber tip coupling, waveguide coupling, and tapered fiber coupling. This method has a higher coupling efficiency compared to other techniques. 

### 3.4. Tapered Fiber Coupling

Tapering can be performed using chemical etching, flame brushing, or drawing from bulk. The tapering process can be applied to single-mode or multimode fiber. The thickness of the tapered fiber can be used to fine-tune the fiber mode’s propagation constant. The tapered fiber must be at a 0 nm, it must also be straight and perpendicular to the WGM surface. Thus, the construction and packaging of tapered coupled WGM elements are challenging [19,20]. Tapered fiber coupling showed the highest coupling efficiency of 99%. Tapering is a relatively simple process. At smaller diameters, the fiber can easily break due to pressure. The fragility of tapered fiber is addressed using LPG structures [21] or multimode fibers [22].

### 3.5. Waveguide Coupling

Waveguides can couple evanescent waves into WGM elements. The waveguide’s evanescent mode must reach the WGM resonator. These systems usually have an input port, a drop port, and a WGM resonator. Light from the input port couples into the WGM resonator. Then, this light cycles in the WGM resonator. This technique has the advantages of flexibility, good quality factors, and integration into compact systems. The WGM and the waveguides can be fully integrated and fabricated on the same substrate. This method needs careful alignment [14].

### 3.6. Prism Coupling

Coupling evanescent waves into a WGM using a prism is one of the earliest coupling methods [23]. A light beam goes through total internal reflection (TIR) inside the prism. Phase-matching is reached by varying the incidence angle. The resulting phase-matched evanescent mode at the interface of the prism and the medium can be coupled into a WGM element [24].

This system can be bulky and has a coupling efficiency of 80% [25]. If phase matching is maintained, prism coupling can achieve single-mode coupling easily compared to other evanescent mode coupling methods. This method is versatile. However, proper alignment is challenging. If the prism’s size is close to the resonators’ size, the coupling can be viewed as waveguide coupling [17].

### 3.7. Fiber-Tip Coupling

Fiber tips can couple light WGM elements. If the fiber tip is smooth at a specific inclination, the light can have a similar phase and propagation constant to the resonance mode of the WGM resonator. The smooth fiber tip surface causes the light to undergo total internal reflection. The fiber tip acts as the prism in a prism coupling. With the usage of fiber, the system becomes more compact [15].

Each previously discussed method has its advantages and disadvantages, summarized in Table 1. Tapered fiber coupling is one of the most efficient and cost-effective coupling methods.

## 4. MBR Fabrication

### 4.1. Two-Step Method (Heat-and-Pull)

Forming MBR elements from regular optical fibers by pinching two close regions with heat or laser to form a bulging region in the middle is commonly called the heat-and-pull method. A CO_2_ laser or a motor-controlled travelling flame heats the fiber locally while the machine pulls the fiber slowly. This step creates a small, tapered region with a homogenous diameter, forming a bulging bottle-like shape between the two tapered fiber regions.

An MBR with a maximum diameter of 3.36 μm and free space coupling showed a quality factor of around 1000 [29]. A heat-and-pull machine was structured to produce bottle diameters of 12 μm [30]. Heat-and-pull MBRs coated with Silicon nano-crystals showed a Q factor of up to 10^6^ [31].This method requires careful planning and handling but can be used to form a wide range of MBRs.

### 4.2. Soften-and-Compress

**Figure 3:**
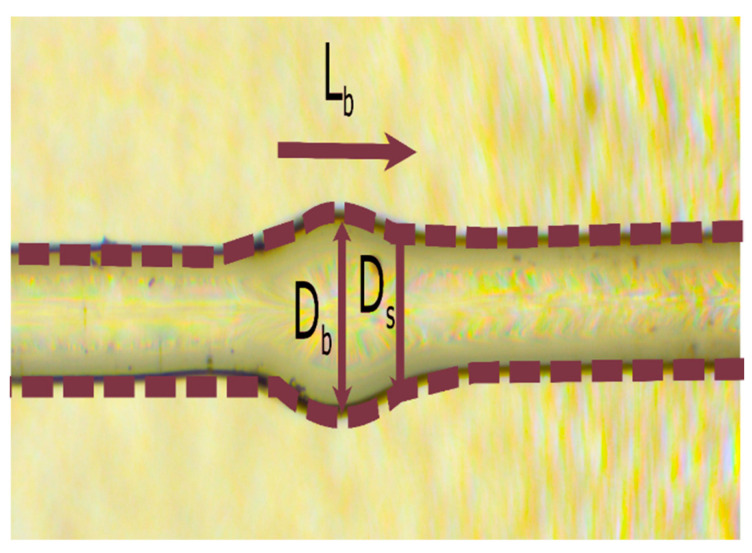
A microbottle resonator formed by soften-and-compress technique with a bottle diameter (*D*_*b*_), stem diameter (*Ds*), and the bottle length (L_b_).

Soften-and-compress is a simple method that uses a fusion splicer on an intact fiber. Splicing is the mechanical–thermal method of fusing two cleaved fibers together. The two fiber ends are pushed towards each other while heat is applied until the fiber ends soften and fuse. Resistive coils or arc discharges can be used to heat the fiber ends. Figure 3 shows an MBR built by this method with.

Splicing machine arcs are applied to an intact fiber in the soften-and-compress technique. The arc heat causes the fiber to soften, and the machine compresses the fiber. The arcs can be applied as often as needed to reach the bottle diameter. A microbottle diameter of 250 μm was reached with this method. Q factors of up to 10^7^ [32]. Splicing machines are cost-effective equipment. However, the fiber should be secured well inside the splicing machine to ensure a symmetric MBR shape [32,33,34].

### 4.3. Polymer Self-Assembly

**Figure 4:**
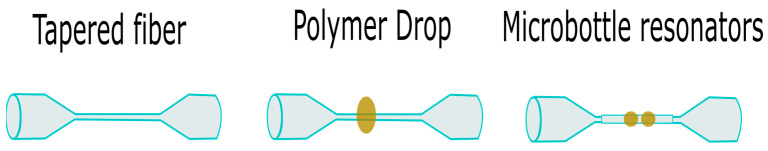
Self-assembled MBR.

This process is non-mechanical and has two main steps: creating liquid bottle-shaped bulges on fiber using interfacial tension and curing the droplets to solidify using heat or UV light. A drop of the adhesive polymer is placed on a vertically tapered fiber as shown in Figure 4. This drop will fall due to gravity. However, some of that polymer adheres to the fiber and forms several bottle-shaped bulges on the tapered or regular fiber. Depending on the choice of polymer, sometimes these MBR need heat or UV curing to solidify fully. The size of the MBR can be controlled by controlling the tapered fiber’s diameter or the choice of adhesive material. 

Norland Optical Adhesive 61 (NOA-61), a UV-curable adhesive, was used to form an MBR with L_b_ 140 μm, D_b_ 66 μm, and D_s_ 38 μm. Q factors can reach 10^5^. NOA61 is particularly suitable for self-assembly due to the surface energy difference between NOA61 and the silica of the fiber. This difference is more than 10 dynes/cm, which is convenient for contact and wetting. When the NOA61 adhesive is dropped on the tapered fiber, the adhesive force causes the NOA61 to wet the surface of the tapered fiber, and the cohesive force of NOA61 causes the droplet to form a ball on the surface to minimize contact with the silica. The effect of both the adhesive force and cohesive force, commonly called interfacial tension, forms the MBR bulges on the surface of the tapered fiber [35].

Polydimethylsiloxane (PDMS) was used to form an MBR on a tapered fiber using the self-assembly method. The PMDS droplet was cured with heat for 40 minutes to form the MBR. The PMDs MBR had a maximum diameter of 480 μm, a neck-to-neck distance of 906 μm, and a Q factor close to 10^4^ when coupled with tapered fiber [36].A gas sensor was built using the self-assembly of Polydimethylsiloxane (PDMS) on a tapered fiber. The tapered fiber was repeatedly dipped in the PMDs material. This process was used to obtain MBRs with diameters ranging from 98 to 213 μm [37].

PMMA-based MBRs were self-assembled on a tapered fiber and used for temperature sensing. These PMMA MBRs were doped with cadmium selenide quantum dots (QDs). The MBR diameters were 5–6 µm. The quality factor of these PMMA MBRs was >10^3^ [38].

SU-8′s UV curing time is relatively short compared to PMMA and PMDS. An MBR formed with a SU-8 drop on a 125 μm diameter optical fiber and coupled with a SU-8-coated waveguide had a Q factor of $\sim 10^{4}$ with diameters ranging from 300 to 564 μm [39]. For lasing applications, dye-doped SU-8 MBRs had diameters as small as 5–10 μm with a passive Q factor above 10^5^ [2]. Resin materials, such as NOA61, NOA63, and NOA81, were also used to form MBR. For instance, an NOA81 MBR with a bottle diameter of 512 μm showed a Q factor of $\sim 8\times 10^{4}$ when coupled via a waveguide [40].

This process can be optimized using cost-efficient polymers. The polymers used can be doped with nanoparticles to produce specific sensors. This method shows great promise for forming MBRs for many sensing applications.

## 5. MBR Sensor

MBRs’ high Q factor and small mode volume makes them a good choice for sensing applications. MBR sensors can be environmental, chemical, or biological. For instance, hollow core silica capillary MBRs are used for magnetic field sensing and showed an 8.45 pm/Gs sensitivity with a Q factor of 1.28 × 10^6^ [41]. A packaged optofluidic MBR for fluid flow rate sensing showed maximum sensitivity of 0.079 pm/(μL/min) within 0–200 μL [42]. However, hollow MBRs sensors can become fragile and challenging.

Solid-state MBR sensors have the analyte on the outer medium. A hollow MBR can have the analyte inside it or the MBR. The sensing principle of MBR is to measure changes in the resonance wavelength shift or power output variations in the presence of the analyte. MBRs can be used in many sensing and measurement applications. The following section summarizes chemical, temperature, and relative humidity MBR experimental sensors.

### 5.1. MBR Sensing Parameters

#### 5.1.1. Sensitivity

Sensitivity is the ratio of the wavelength shift to the measured analyte change. For example, if the analyte is relative humidity, the sensitivity can be calculated as the total resonance wavelength shift divided by the total relative humidity change: Δλ/ΔRH. Larger quality factors show MBRs’ potential to be responsive to changes in their surrounding medium. However, noise sources control the MBR detection limits. Noise in a sensing system can happen due to light source instability, laser instability, or human errors. These limitations are technical and can be overcome. However, other noise sources are fundamental, such as the quantum nature of light and shot noise [43].

#### 5.1.2. Time Resolution

For MBRs, the best time resolution is approximately the resonator photon lifetime or response time $\tau =\gamma^{-1}$ [43]. Higher quality factors limit measurements on time scales smaller than the resonator photon lifetime. Thus, a trade-off exists between sensitivity and time resolution [43]. For practical sensing applications, the modulation bandwidth of the laser light source limits the time resolution. Using a frequency-modulated light source can help improve the time resolution. Other equipment, such as optical spectrum analysers, can also limit the time resolution to milliseconds [44].

#### 5.1.3. Specificity

Sensors, ideally, show a response to their intended analyte only. An MBR sensor’s specificity can depend on the resonator’s material. The material can be glass or polymer. Materials attached to the surface of the MBR improve specificity. For instance, hydrophilic polymer materials can be used for humidity and moisture detection. For chemical sensors, films and aptamers can improve specificity. For biological sensors, immobilizing virus proteins, antibodies, or aptamers can be used to capture only the intended analyte.

Due to the fouling effect, the medium in which the sensor is deployed also plays a role in specificity. Fouling is the absorption of non-specific molecules from the surrounding medium. Fouling is less likely for humidity, temperature, and gas sensors than for bio-analytes such as blood [44]. Additionally, the linearity of the sensor determines the response of the sensor output. The long-term stability of the sensor response is also vital.

### 5.2. MBR Temperature Sensor

MBR’s resonance wavelength can shift with temperature changes. Thermal expansion and thermo-optic effects can affect the properties of MBRs. The thermal expansion coefficient is the change in a material’s dimension with temperature changes. The thermal expansion coefficient can be positive (expansion), negative (contraction), or zero. The thermo-optics effect changes a material’s refractive index with temperature changes. Therefore, temperature changes in the MBR surrounding environment can affect the fiber radius and refractive index, causing the MBR resonance wavelength to shift with temperature changes.

Simulations conducted on the effect of temperature changes on an MBR with a 500 length at temperatures of 200, 300, and 400 K showed the sensitivity of the MBR to temperature increased with larger radii in the range of 10–100 μm. In addition, the sensitivity of a 100 μm MBR reached 10 pm/K [45].

Lab experiments conducted on MBR temperature sensors coupled with a tapered fiber showed a sensitivity of 0.0149 dB/C and a wavelength shift of 1.3 pm/°C in the temperature range of 40–100 °C. The MBR was formed using the soften-and-compress technique with a bottle diameter (*D*_*b*_) of 190 μm, stem diameter (*D*_*s*_) of 125 μm, and the bottle length of 182 μm. This sensor had a Q factor, and a 2 μm tapered fiber coupled light into it [46].

A 3D packaged MBR sensor showed a sensitivity of 10.5 pm/K and a Q factor close to 10^6^. The soften-and-compress technique was also used to form this MBR. The central diameter was 207 μm, and a tapered fiber coupled 1550 nm light into the MBR. The 3D packaging helped secure the MBR and tapered fiber [47].

MBRs embedded in isopropanol showed a temperature sensitivity of 61 pm/°C. The sensing setup used isopropanol’s high thermal refraction coefficient to achieve sensing in a limited range of temperatures of 20–40°C [48].

Self-assembled PMMA MBRs doped with cadmium selenide quantum dots (QDs) had a 9.2 pm/C sensitivity. The observed Q factor for this MBR was 5 × 10^3^. This structure was free-space coupled [38].

### 5.3. MBR Humidity Sensors

MBR surfaces can be affected by changes in relative humidity in the surrounding environment. The water molecules in the air affect the refractive index of the MBR, causing a shift in the resonance wavelength and affecting the output power of the sensors.

A self-assembled MBR made with (Locatite3525)/graphene oxide showed a 0.161 nm/%RH sensitivity in the 22–81% range. High-temperature annealing reduced the sensor’s temperature effects [49]. The soften-and-compress technique formed an MBR with a D_b_ = 190μm, stem diameter of D_s_ = 125 μm, and bottle length of L_b_ = 182 μm. This structure showed a 0.0487 dB/% sensitivity in the relative humidity range of 40–80%, and the Q factor of this structure was 10^4^. A two-tapered fiber coupled the light into this MBR [50].

In addition, the effect of using multiple MBR elements was investigated. The same tapered fiber was coupled to two MBRs. Then, the structure performance as a relative humidity sensor was studied. In that experiment, the single MBR structure with the largest diameter of 190 showed the highest sensitivity of 0.06 dBm/% [51].

Coated MBRs also measure relative humidity. Agarose-coated 190 μm MBR formed by the soften-and-compress method showed a 0.107 dBm/% sensitivity in the 35–85% RH relative humidity range. The agarose gel was a 0.5% solution. In addition, 1% agarose gel was used to coat a similar MBR structure, which showed a sensitivity of 0.0899 dB/RH% [52,53]. HEC/PVDF-coated MBR had a 0.111 dB/RH% sensitivity in the 40–80%RH relative humidity range [54].

### 5.4. MBR for Chemical Sensing

MBR resonance wavelength can be affected by changes in the refractive index of the surrounding medium. A chemical solution can change the refractive index of the surrounding medium and cause a shift in the resonance wavelength or changes in the power output of the MBR sensor. A self-assembled MBR made with single-mode fiber (SMF), palladium-tungsten trioxide (Pd-WO3), and polydimethylsiloxane (PDMS) was used for hydrogen detection and showed a −3.091 nm/% sensitivity at 25 °C [55].

MBR sensors detected formaldehyde in the concentration range of 1–5%. This setup showed a sensitivity of 4.397 dB/%. The soften and compress technique formed an MBR with a 190 m diameter. A two-tapered fiber coupled the light into the MBR [56]. The tapering diameter of the fiber coupling light into an MBR with similar dimensions and setup was investigated in another work. An eight-tapered coupling fiber had better sensitivity to formaldehyde solutions compared to a ten-tapered fiber [57].

An ethanol sensor was built using an MBR with a bottle diameter of 170 μm and showed a 0.1756 dB/%ppm sensitivity in the 10–100% ethanol concentration range. The soften-and-compress technique was used to shape the fiber [58]. A PVA-coated MBR was used for ethanol sensing and showed a 0.2699 dB/%ppm and 0.2 pm/%ppm sensitivity [59].

A sodium hypochlorite sensor was built using a similar setup of soften-and-compress MBR with tapered fiber coupling. It had a Q factor $>10^{5}$ and a 180 μm bottle diameter. This sensor showed a sensitivity of 0.002 nm/%ppm and 3.7319 dB/%ppm in the 1–6% hypochlorite concentration range. It showed a resonance wavelength shift of 1552.203 nm to 1552.213 nm in the same sodium hypochlorite concentration range [60]. 

Table 2 summarizes the performance parameters of recently developed MBRs for chemical, temperature, and relative humidity sensing.

## 6. Conclusions

Microbottle resonators (MBRs) have high Q factors and small mode volumes, which makes them ideal for sensing applications. In this paper, the optical properties of MBRs, the light coupling methods, and the sensing principles are discussed. MBRs fabrication methods and their use in sensing applications are also discussed. MBRs can be used in sensing applications due to the versatility of their fabrication methods. Single-particle detection and biomedical applications can be a future mode of direction for the MBR sensors.

## Figures and Tables

**Table 1:** MBR coupling method comparison.

Coupling Methods	Coupling Efficiency	Advantages	Disadvantages	Ref
Free space	20% and up to 30%	Versatility	Low coupling efficiency; interference and difficult maintenance can be an issue	[18]
Prism	80%	Convenient handling	Larger size, challenging integration	[23,26]
Fiber tip	60%	Small size, easy integration	Challenging fabrication and packaging; fiber tip can be fragile	[11,17,27]
Tapered fiber	>99%	The highest coupling efficiency; simple, well-established fabrication methods Easy integration; cost-effective	Tapered fiber can be fragile; packaging can be challenging	[16,19,28]

**Table 2:** Sensing performance of experimental MBR setups.

Analyte	Structure	Sensitivity	Ref
Temperature	MBRs embedded in isopropanol	61 pm/°C	[48]
Temperature	3D packaged MBR	10.5 pm/K	[47]
Temperature	Silica MBR	0.0149 dB/C–1.3 pm/°C	[46]
Temperature	Self-assembled PMMA MBRs doped with cadmium selenide quantum dots	9.2 pm/°C	[38]
Humidity	Agarose coated MBR	0.107 dBm/%	[52]
Humidity	self-assembled (Locatite3525)/graphene oxide	0.161 nm/%RH	[49]
Humidity	Silica MBRs	0.0487 dB/%	[50]
Humidity	HEC/PVDF coated MBRs	0.111 dB/RH%	[54]
Hydrogen	Self-assembled (SMF)/(Pd-WO3)/(PDMS)	−3.091 nm/%	[55]
Formaldehyde	Silica MBRs	4.397 dB/%.	[56]
Ethanol	Silica MBRs	0.1756 dB/%ppm	[58]
Ethanol	PVA coated MBR	0.2699 dB/%ppm–0.2 pm/%ppm	[59]
Sodium hypochlorite	Silica MBR	of 0.002 nm/%ppm–3.7319 dB/%ppm	[60]
Sodium hypochlorite	PVA coated MBR	5.5176 dB/%ppm–2.4 pm/%ppm	[61]
Magnetic field	Hollow core capillary	8.45 pm/Gs	[41]

## Data Availability

Not applicable.
